# A Three-Dimensional Movement Analysis of the Spike in Fistball

**DOI:** 10.3390/sports4040055

**Published:** 2016-12-02

**Authors:** Andreas Bund, Saeed Ghorbani, Franziska Rathjens

**Affiliations:** 1 Institute of Applied Educational Sciences, University of Luxembourg, Esch-s.-Alzette L-4365, Luxembourg, Luxembourg; 2 Department of Physical Education and Sport Science, Aliabad Katoul Branch, Islamic Azad University, Aliabad Katoul, Iran; s.ghorbani@aliabadiau.ac.ir; 3 Institute of Sport Science, University of Oldenburg, Oldenburg D-26129, Germany; franziska_rathjens@gmx.de

**Keywords:** movement analysis, biomechanics, fistball

## Abstract

Due to its relevancy to point scoring, the spike is considered as one of the most important skills in fistball. Biomechanical analyses of this sport are very rare. In the present study, we performed a three-dimensional kinematic analysis of the fistball spike, which helps to specify performance parameters on a descriptive level. Recorded by four synchronized cameras (120 Hz) and linked to the motion capture software Simi Motion^®^ 5.0, three female fistball players of the second German league (24–26 years, 1.63–1.69 m) performed several spikes under standardized conditions. Results show that the segment velocities of the arm reached their maximum successively from proximal to distal, following the principle of temporal coordination of single impulses. The wrist shows maximum speed when the fist hits the ball. The elbow joint angle performs a rapid transition from a strong flexion to a (almost) full extension; however, the extension is completed after the moment of ball impact. In contrast, the shoulder joint angle increases almost linearly until the fistball contact and decreases afterward. The findings can be used to optimize the training of the spike.

## 1. Introduction

Fistball is a traditional ball game in which two teams with five players play against each other on a field separated by a net. Similar to volleyball, the idea of the game is to hit the ball with a part of the arm or the fist in such a way across the net that it is difficult or impossible for the other team to return it. Within the team, the ball can be played directly or indirectly by three different players, thus, the standard sequence consists of three elements: defense (trapping the ball), passing and offense (attacking shot/spike). Fistball is played mainly in Europe and South America, but is becoming more and more popular in Africa, Asia and North America too. At the World Games as well as at Continental Championships, national teams play against each other. The organization of these events is done by the International Fistball Association (IFA) [1], which was co-founded in 1960 by Germany, Brazil, Switzerland, Austria and Italy.

In recent years, an increasing professionalization on the athletic and organizational levels can be observed. The field of sport science, however, has paid little attention to fistball so far and very few scientific papers on the topic are available. Besides older publications on methodological aspects (e.g., [2,3]), studies often deal with the risk of injury in fistball [4,5]. Runer et al. [5], for example, found over the course of 12 months an overall injury rate of 12.2 injuries/1000 h of exposure. The most common types of injury were abrasions and contusions. An exception is provided by Leser [6], who has developed and evaluated a computer-based observation system for fistball.

Of particular interest to the present work is a study of Soeser and Schwameder [7]. Focusing on the hitting side of the body, they performed a three-dimensional kinematic analysis of the fistball serve. The results can be summarized as follows: (1) The execution of the serve shows in many characteristics (e.g., ball drop, turning back the hitting side shoulder, and body extension in the moment of ball impact) a high degree of standardization, i.e., the execution is very similar across different players and performance levels; (2) A kinematic chain can be seen that typically occurs in throwing movements, i.e., hip, shoulder, elbow and wrist/fist successively reach their maximum speed and become slowed down in order to transfer and maximize the impulses from proximal to distal body segments. As expected, players from the national team reached significantly higher body segments and ball velocities then players from federal or regional leagues.

Similar to the serve, the spike is a typical hitting movement whose main phase is performed above the head. It is therefore expected that the results of Soeser and Schwameder [7]—e.g., in terms of the kinematic chain and the temporal coordination of impulses—are also valid for the spike. However, it should be noted that the spike starts with a one-legged kick-off jump in the air and, thus, is performed without ground contact. Consequently, the biomechanical conditions of serve and spike differ significantly. In addition, the spike usually coordinates with the trajectory of the passed ball and therefore has to be available and executed under highly variable conditions. Generally speaking, a good technical execution of the spike requires that the player has the right distance to the ball and that he or she is able to accelerate strongly and temporally coordinate the segments of the hitting side of his or her body. At the moment of ball impact, speed and trajectory of the ball are determined by the velocity, rotation and inclination of the fist, the point of ball contact, and the movement direction of the hitting arm [7].

The lack of biomechanical and especially kinematic analyses of motor skills used in fistball was the reason to conduct this study. The following aims were determined:

(1) The description of the spike in fistball based on three-dimensional recorded kinematic parameters with high frequency.
(2) The identification and weighting of performance-determining factors by a comparative analysis of kinematic parameters of different players.

Unlike Soeser and Schwameder [7], both sides of the body were included in the analysis.

## 2. Method

### 2.1. Participants and Task

Three female fistball players aged from 24 to 26 years old who play in clubs in Germany’s 2nd league on the attack position took part in this study. The players were right-handed, 1.63 to 1.69 m tall and weighed at the time of the study from 55 to 72 kg. The task was to perform from a short run-out a spike against a fist ball attached to a rope from the ceiling, just above the fistball net that was stretched to a height of 1.90 following the competition rules. The ball height varied slightly depending on the body size of the player and was previously determined in several trials. Table 1 shows the anthropometric measures of the players as well as the individual ball heights. The procedure of the study was designed according to the guidelines of the Helsinki declaration. All players provided informed consent prior to the study.

### 2.2. Data Recording

**Figure 1:**
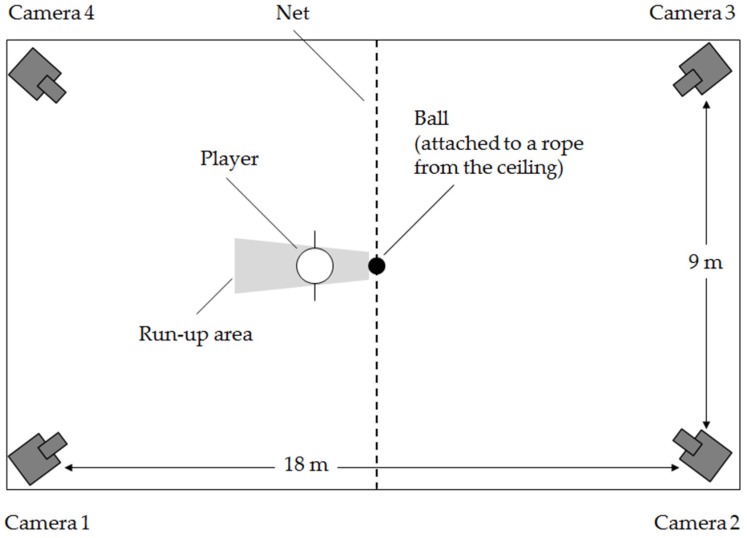
Measurement setup.

The spike (including the run-out) was recorded by four synchronized and mutually perpendicular positioned cameras on 1.65 m high tripods (Basler Scout) with a frame rate of 120 Hz. Based on the dimensions of a volleyball court, four cameras with an oblique view filmed the player, two cameras from the front and two from the back. The recorded space was calibrated using a cube with 2 × 2 × 2.5 m. Figure 1 shows the exact position of the cameras and the distances among each other. Recordings were made in the gymnasium of the University of Oldenburg (Germany). Each player completed 4 to 6 trials hitting the ball in a dynamic and fluent movement. For the subsequent kinematic analysis, a total of 7 body points of the players (foot, ankle, knee, hip, elbow, shoulder, and wrist) were bilaterally covered with light-reflective markers. Five of these markers were affixed directly to the skin, only the foot and the hip markers were placed on the shoe and a tight-fitting shirt, respectively. The exact localization of the markers is described in Table 2.

### 2.3. Data Processing

The trajectories of the above-mentioned body points were automatically digitized using the motion capture software Simi Motion 5.0 (Simi Reality Motion Systems, Unterschleißheim, Germany). The beginning of the third last step (ground contact) was defined as starting point of the movement; the seventh frame after the ball contact was considered as the end point. In order to compare the recordings of the various trials and players, they were standardized in the post-processing on each 100 frames exactly. Specifically, the following kinematic parameters were calculated:

- Path velocities of the hip, shoulder, elbow and wrist (unilateral, for the hitting side of the body)
- Joint angles of elbow, shoulder (unilateral, for the hitting side of the body), knee and hip (bilateral)
- Height of the hip and shoulder (bilateral)
- Center of gravity (CoG)

**Figure 2:**
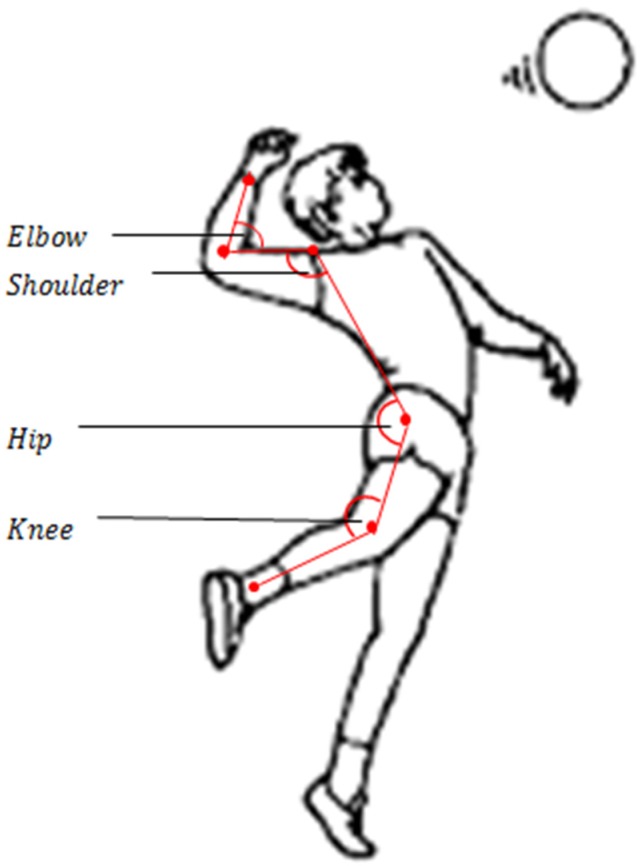
Joint angles calculated in the study.

The temporal coordination of path velocities and accelerations of the hip, elbow, shoulder and wrist of the hitting side of the body is pivotal to the impulse transfer from the wrist to the ball and largely determine its speed. The joint angles were calculated in the clockwise direction through the Simi Motion software by applying Equation (1) on each single frame. The basis was the *x*-coordinates of the body points, that is, the angles were analyzed in the sagittal plane. The elbow joint angle was determined by the elbow as apex and the wrist and shoulder as end points of the corresponding triangle-legs. The shoulder joint angle was defined by the shoulder as apex and the elbow and hip as end points. The hip angle was measured with the hip as apex and the shoulder and knee as end points. Finally, the knee was used as apex and the ankle and hip as end points to determine the knee joint angle. Figure 2 demonstrates the joint angles and their corresponding vectors.

joint angle = acos(scalar product(v1, v2)/(norm(v1) × norm(v2)))(1)

The hip and shoulder heights were calculated from the *z*-coordinates, i.e., in the frontal plane, and indicate the players posture during run-up, take-off and hitting phases. The assessment of the CoG was made by the Simi Motion software according to the Hanavan human body model. The CoG parameter was used to determine the run-up velocity (horizontal CoG-velocity before the take-off) and the jump height (maximum CoG-height in flight phase—CoG-height at last ground contact). Finally, the initial path velocity of the ball was calculated as a key parameter of the spike movement.

## 3. Results

### 3.1. Kinematic Description of the Spike

**Figure 3:**
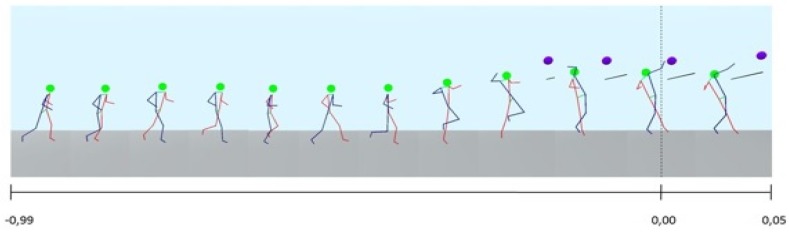
Kinegram of the fistball spike (player 1), with timeline (s). higher resolution picture. Comma should be changed into dot.

Figure 3 shows as an example the kinegram of player 1. The sequence shows how the player performs the run-up, the take-off, the backwards torsion of shoulder and arm on the hitting side of the body, the subsequent acceleration of these body segments in the direction of the ball, the ball impact with simultaneous body extension and the preparation of the landing. The run-up velocities differ only minimally and range for all players from 3.31 m/s to 3.49 m/s. The maximum jump height varies between 0.32 m and 0.43 m. The time intervals between take-off, ball impact and landing are again relatively stable; they range from 0.26 s to 0.16 s and 0.35 s to 0.20 s, respectively. Thus, similar to the fistball serve [7], we also found for the spike only small inter-individual variance regarding the kinematic of movement phases. However, it should be noted, that the players who participated in this study had a very similar level of performance.

### 3.2. Path Velocities

**Figure 4:**
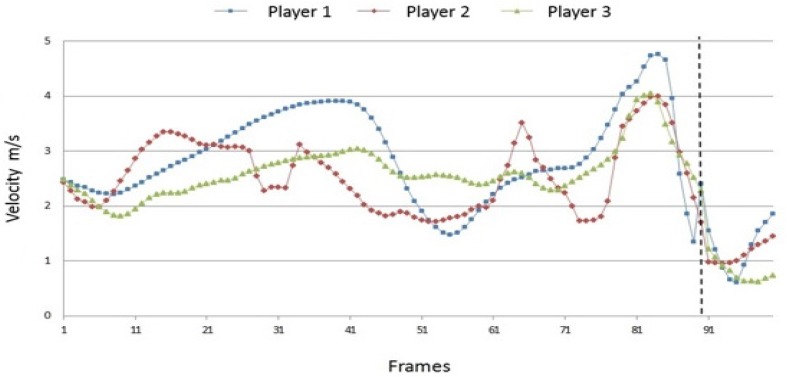
Inter-individual comparison of the path velocities of the right shoulder joint (100 frames; dotted line = ball impact).

**Figure 5:**
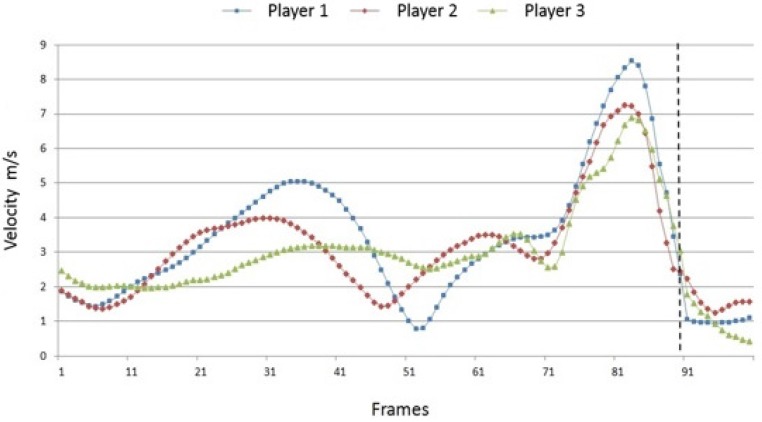
Inter-individual comparison of the path velocities of the right elbow joint (100 frames; dotted line = ball impact).

**Figure 6:**
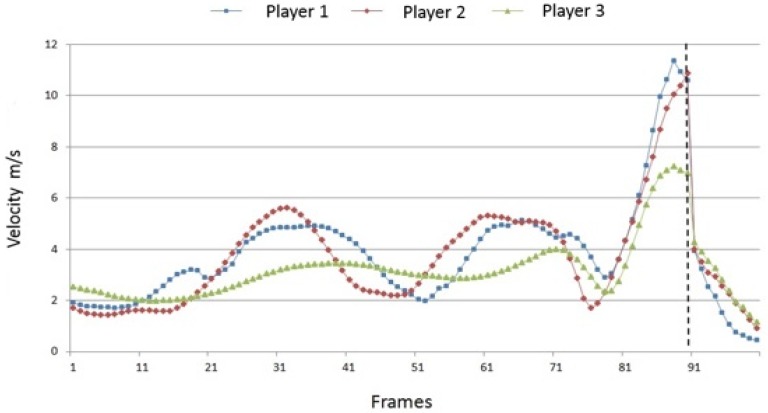
Inter-individual comparison of the path velocities of the right wrist joint (100 frames; dotted line = ball impact).

Figure 4, Figure 5 and Figure 6 show the path velocities of the shoulder, elbow and wrist joint on the hitting side of the body. Aside from inter-individual similarities, the data indicate various individual characteristics of the players’ movement. During run-up, the path velocity of the right shoulder rises and falls according to the individual running cadence of the player. The cycle varies considerably, and the differences between the velocity minima and maxima are significant. Player 1 shows clear differences concerning these parameters. Common to all players is that they rapidly accelerate the shoulder just before the ball impact in order to reach maximum speed. After the impact, the shoulder is strongly slowed down in a short time period.

The path velocities of the elbow and the wrist joint can be described analogous in essential points. During the run-up phase, the path velocity of the elbow again shows a cyclical pattern. Overall, the cycles vary inter-individually less than in the shoulder; however, the minimum–maximum differences are significantly smaller for player 3 than for players 1 and 2. The very fast acceleration of the elbow joint as well as the deceleration after the ball contact are also very similar between the players. Just before the ball impact, the players reach an almost equal velocity maximum. In contrast to this, the speed of the wrist reaches its maximum exactly when the fist hits the ball; before and after that moment a strong positive or negative acceleration can be observed. While running-up towards the net, the path velocity of the wrist again oscillates according to the players’ individual rhythm. As with the elbow joint, these cycles occur “flatter” for player 3 than for players 1 and 2. Furthermore, player 3 does not achieve such a high maximum speed as the other two players.

The velocity maxima of the captured body points and the resulting ball speed are shown in Table 3. For all players, the velocity maxima continuously increase from proximal (hip) to distal (wrist) body points. This data suggests that in this case a kinematic chain is operating, in which the speed maxima of the involved body segments are placed in sequence so that the (distal) end link of the chain experiences maximum acceleration.

**Figure 7:**
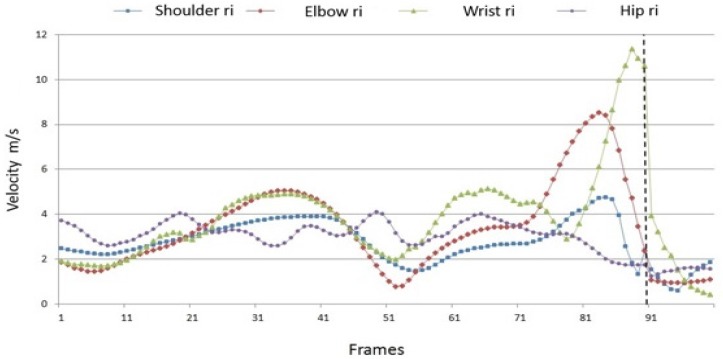
Path velocities of the body points on the hitting side of the body of player 1 (100 frames; dotted line = ball impact).

Of course, the existence of a kinematic chain becomes clearer if the path velocities of the relevant body points are presented together. This is done in Figure 7 using the example of player 1, who achieved the highest ball speed with 12.45 m/s.

### 3.3. Joint Angles

**Figure 8:**
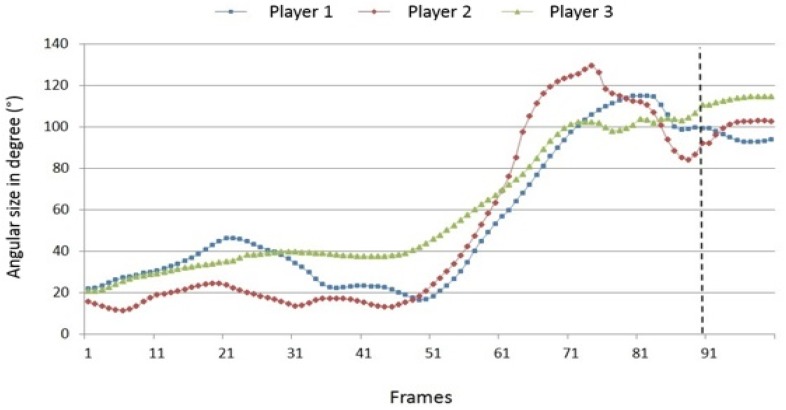
Inter-individual comparison of the joint angles of the right shoulder joint (100 frames; dotted line = ball impact).

**Figure 9:**
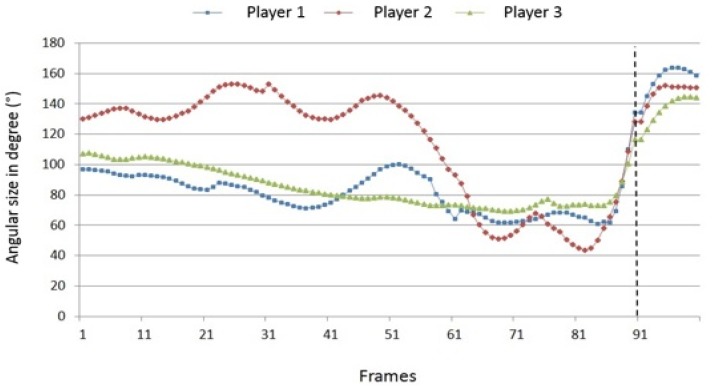
Inter-individual comparison of the joint angles of the right elbow joint (100 frames; dotted line = ball impact).

Compared to the velocity data, the course of shoulder and elbow angles (Figure 8 and Figure 9) clearly reveals greater inter-individual differences. This concerns in particular the players’ run-up and take-off phases. Player 2, for example, keeps her playing (“hitting”) arm during the run-up close to the body so that the shoulder angle slightly opens and closes according to the running cadence. During and after take-off, a very fast extension of the shoulder joint to an angle of ca. 130° takes place, which just as quickly evolves into a strong flexion at the moment of ball contact. For player 1 and especially player 3, the angle course is basically similar, however, the extension and flexion of the shoulder angle occurs in a smaller range and less abruptly. Concerning player 3, Figure 7 illustrates that the playing arm is stretched far out when she is moving toward the net (shoulder angle about 40°). That might be partly responsible for the low jump height of 0.34 cm, because in this position the arm can hardly be used as an additional swinging element. In contrast to the other players, there is no significant flexion of the shoulder angle when she hits the ball. Instead, the hip angle decreases considerably at this moment, thus, player 3 is using the entire upper body to perform the hitting phase of the spike.

In respect to the elbow angle, even greater differences occur between the players, only during and after the ball impact do the profiles tend to match each other. Previously, the arm posture was very specific to the individual and notably had no consistent relation to the run-up and take-off action. Player 1 successively reduces the elbow angle during the run-up phase; only the take-off leads to a temporary extension. Concerning the countermovement of the playing arm, the elbow angle first oscillates slightly followed by an explosive stretching until ball contact; however, the ball contact is made shortly *before* the maximum extension of the arm is reached. In contrast to this, player 2 starts with an extended elbow angle (<120°), which is then gradually flexed on after the take-off and finally opened again to hit the ball. Striking (and certainly unusual) in this case is the short-term (slight) “intermediate” extension during the jumping phase (about frame 75). Finally, player 3 shows a third variant: During run-up and take-off the elbow is bent more and more. The extension of the arm takes place only before and after the ball impact and is a bit less pronounced than for the players 1 and 2.

Are joint angles and path velocities systematically linked with each other, e.g., at the time of ball contact? Higher degrees of extension and, thus, greater body angles should result in higher path velocities of the distal points of the body. Of course, the question cannot be answered reliably with the very small sample used in this study; however, the available data supports this assumption: For example, player 1 hits the ball with an elbow angle of 129° achieving a maximum wrist velocity of 11.79 m/s, while player 3 stretches the elbow only to 115°, which leads to a wrist velocity of only 8.37 m/s.

### 3.4. Height of Hip, Shoulder and CoG

**Figure 10:**
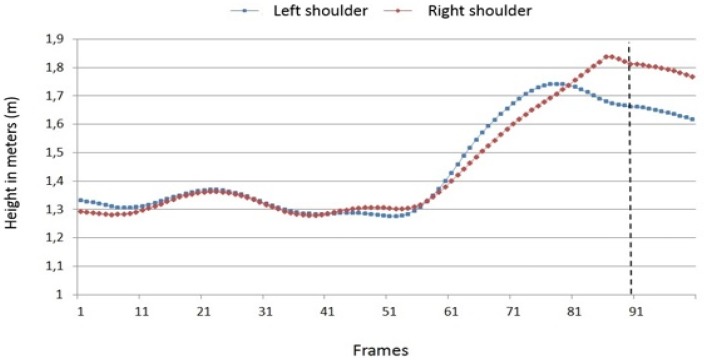
Height profies of right and left shoulder of player 1 (100 frames; dotted line = ball impact).

Overall, the bilateral measurement of the height profiles of hip and shoulder shows only small inter-individual variance: During run-up, hip and shoulder move on both sides at the same level. During the take-off of the player, both pivot points are raised; however, on the (right) hitting side of the body they are raised higher than on the left side due to the increasing extension of this part of the body. At the moment of ball contact, the right hip and shoulder reach the highest point in all players. However, the difference between the height on the right side and the left side of the body vary from player to player. In relation to the hip, for player 1 this was ca. 9 cm, for player 2, ca. 16 cm and for player 3, only ca. 5 cm. In other words, player 2 is clearly more tilted during the hitting phase than the other two players. The height difference between the right and left shoulder is about 16 to 18 cm. Figure 10 illustrates as an example the height profile of the left and right shoulder of player 1.

The measurement of the CoG provides information on the horizontal velocity while running-up and on the jumping height of the players. The run-up velocities just before the take-off are at 3.43 m/s, 3.49 m/s and 3.31 m/s and thus very little. Compared with this, the differences in the jump heights are greater: 0.43 m, 0.32 m and 0.34 m. Due to the sample size, it is not possible to determine a correlation between run-up velocity and jump height. In addition, the CoG height profile is very similar for all players.

## 4. Discussion

Based on the fact that only very little sport science research on fistball is available to date, the aim of the present study was to carry out, on a descriptive level, a three-dimensional kinematic analysis of the spike in fistball. Using camera-based motion capture software (Simi Motion 5.0), kinematic parameters such as velocity, angle and trajectory of different body points were bilaterally recorded, digitized and finally evaluated. Based on the comparison of several players, we identified and weighted parameters that significantly affect the spike performance. The results can be summarized as follows:

The kinematic pattern of movement phases shows only a small range of inter-individual variance. Run-up velocity and temporal sequence of run-up, take-off and landing are relatively similar across the players.

1. The analysis of the path velocities of several body points demonstrates individual styles in the execution of the fistball spike; they concern, in particular, timing and range of velocity changes.
2. In terms of a kinematic chain, the hip, shoulder, elbow and wrist joint reach their maximum speed successively and temporarily coordinated. The deceleration of each more proximal joint causes the impulse to be (partly) transferred to the next joint or limb, so that the wrist, as the end of the chain, receives the highest speed.
3. The courses of the joint angles, especially of that of the hitting arm, differ considerably from player to player. As expected, extended elbow angles tend to be associated with greater speed of distal body points.
4. The height profile of hip, shoulder and CoG is basically similar for all players. Due to the extension during the hitting phase, the points on the hitting side of the body are higher than the corresponding points on the opposite side. The hip axis is clearly more inclined than the shoulder axis.

Overall, our results are in line with those of Soeser and Schwameder [7] on the fistball serve, taking into account that the latter of course found higher values for joint and ball speed due to the gender (male) and level (semi-professional) of their participants (e.g., ball speed: 24.69–28.01 m/s compared to 12.19–12.69 m/s in our study). The impulse transfer within a kinematic chain was clearly demonstrated in both studies. It occurs typically in such throwing movements. As expected, fistball is, in this regard, comparable to other sports in which an object has to be maximally accelerated by a strike (short contact) or a throw (long contact): the biomechanical principle of optimal coordination of impulses applies [8]. Specifically, this is also documented for the spike in volleyball [9], the goal shot in handball [10,11,12], and the tennis serve [13]. Another commonality between the present study and that of [7] is the relatively strong lateral inclination of the upper body during the hitting phase, which is reflected in significant height differences between left and right hips and shoulders.

In connection with the coordination of impulses, positive relationships between certain parameters are to be expected, firstly between path velocities and resulting ball speed, and secondly, between joint angles and path velocities. In both cases, our findings indicate such a positive relationship; however, it is not supported by statistical evidence because of the small sample size. This reflects a serious weakness of the present study: The comparative analysis had to remain on the descriptive level; an inferential statistical analysis was not possible. A larger sample consisting of players of different performance levels, as used by Soeser and Schwameder [7], would have been desirable in order to get statistical evidence about the relationship of various kinematic parameters and their influence on the fistball spike performance. Another problem comes from the limited number of body points that were included in the analysis. Although we have measured the trajectories of the main joint angles on both sides of the body, some important aspects of the spike movement were not considered. For example, the prono-supination of the hitting arm—i.e., the internal-external rotation of the arm during the execution of the spike—was not taken into account. Neither was the angle between the player’s body and the hall floor as a measure for the body inclination before and during the ball contact determined. Our analysis is therefore not complete and in terms of reliability the results should be seen as an approximation.

## 5. Conclusions

Nevertheless, we believe that from the kinematic description done in this study valuable insights for training and competition arise, especially as the analysis—apart from the ball passing—was carried out under typical game conditions. It has become clear, for example, how important it is to temporally coordinate the single impulses during the hitting phase in order to maximize the terminal speed of wrist and ball. Therefore, during practice, special attention should be taken that elbow, shoulder and wrist are accelerated in quick sequence, one after the other. Since the first impulse for the spike is produced in the torso, it seems reasonable to consider strengthening exercises for the core muscles as a regular part of the training regime. Vigorous torso and shoulder muscles can also help to prevent muscle injuries, which are quite common in fistball due to the partially strong inclinations and torsions of the upper body [4] (as demonstrated in this study.) Electromyography methods would provide further insight in this case. As the run-up is usually very short, the run-up velocity is believed to play only a minor part in the jump height. The jump height is mainly the result of the explosive strength of the legs and the coordinative quality of the jumping motion (e.g., fluent connection of up and downward movement, use of initial force, and arm movement). In addition, the run-up in fistball is performed less in the up-front direction than, for example, in Handball. Of course, dynamometric measurements using force plates would be interesting in this context; combined with our findings, they would lead to a better understanding of the biomechanical structure of the fistball spike.

The progressive professionalization in fistball and the increasing popularity of this sport in many countries should be reflected in the future by a stronger interest from sport science. This study as well as the work of [4,5,6,7] are a starting point.

## Figures and Tables

**Table 1:** Anthropemetric measures of the participants and individual ball heights.

Participant	Body Weight (kg)	Body Height (m)	Ball Height (m)
Player 1	55	1.62	1.94
Player 2	63	1.64	1.96
Player 3	72	1.69	1.96

**Table 2:** Body points included in the kinematic analysis.

Body Point	Body Side	Localization
Foot	bilaterally	Basis Metatarsales V
Ankle	bilaterally	Malleolus lateralis
Knee	bilaterally	Epicondylus lateralis femoris
Hip	bilaterally	Trochanter major
Elbow	bilaterally	Epicondylus lateralis humeri
Shoulder	bilaterally	Acromio-clavicular join
Wrist	bilaterally	Os capitatum

**Table 3:** Maxima of path velocities and resulting speed of the ball (m/s).

Title	Hip	Shoulder	Elbow	Wrist	Ball
Player 1	4.41	5.23	8.98	11.79	12.45
Player 2	4.22	4.65	8.28	11.43	12.19
Player 3	3.82	4.95	8.37	8.65	10.89
